# Curcumin Attenuates Decidual Stromal Cell Ferroptosis and Restores Impaired Decidualization in Recurrent Spontaneous Abortion by Targeting BRD4

**DOI:** 10.1002/fsn3.72205

**Published:** 2026-08-03

**Authors:** Yiming Ma, Weiping Chen, Tian Xie, Yueling Wu, Yangyang Xing, Xiaoxuan Zhao, Jialin He

**Affiliations:** ^1^ Faculty of Chinese Medicine and State Key Laboratory of Mechanism and Quality of Chinese Medicine Macau University of Science and Technology Macau China; ^2^ College of Pharmacy Hangzhou Normal University Hangzhou China; ^3^ Zhuhai M.U.S.T. Science and Technology Research Institute Guangdong‐Macao In‐Depth Cooperation Zone in Hengqin Zhuhai China; ^4^ Department of Traditional Chinese Medicine (TCM) Gynecology Hangzhou TCM Hospital Affiliated to Zhejiang Chinese Medical University Hangzhou China

**Keywords:** BRD4, curcumin, decidualization, ferroptosis, recurrent spontaneous abortion

## Abstract

Recurrent spontaneous abortion (RSA) is a major reproductive health challenge with limited clinical options. Curcumin, a natural polyphenol widely consumed as a dietary supplement, has shown promise in improving pregnancy outcomes; however, its specific molecular targets remain obscure due to its broad bioactivity. This study aimed to define curcumin's impact on RSA and identify its direct molecular target to provide a mechanistic basis for its nutritional application. We employed an integrated approach combining phenotypic analysis in the RSA mouse model and target discovery in human endometrial stromal cells. Target identification was performed using unbiased Limited Proteolysis‐Mass Spectrometry (LiP‐MS). The interaction between curcumin and the identified target, BRD4, was confirmed through molecular docking, dynamics simulations, functional genetics, and chromatin immunoprecipitation (ChIP‐qPCR). Functional outcomes were assessed by measuring key ferroptosis markers and mitochondrial morphology using transmission electron microscopy. Decidualization was analyzed in both mouse tissues and cells subjected to a decidualization and ferroptosis induction protocol. Curcumin significantly reduced embryo resorption and restored decidual morphology and marker expression (PRL and IGFBP1) in RSA mice. LiP‐MS analysis in human decidual stromal cells identified ferroptosis as the primary pathway targeted by curcumin. Both in vivo and in vitro validation showed that curcumin inhibits ferroptosis, decreasing lipid peroxidation, restoring glutathione balance, and preserving mitochondrial integrity, which in turn rescued decidual marker expression. Proteomic integration with ferroptosis databases identified BRD4 as a central hub. Mechanistically, curcumin binds BRD4, thereby suppressing BRD4‐driven expression of TFRC and ACSL4, which blocks ferroptosis and rescues decidual markers. This research uncovers a BRD4‐driven ferroptosis pathway as a key pathogenic mechanism in RSA. Our results demonstrate that curcumin acts as an epigenetic modulator by directly targeting BRD4, thereby suppressing this pro‐ferroptotic transcriptional program. These findings provide critical mechanistic evidence supporting curcumin as an evidence‐based nutritional intervention for RSA.

## Graphical abstract

Curcumin attenuates decidual ferroptosis and restores decidualization by targeting BRD4 in RSA.

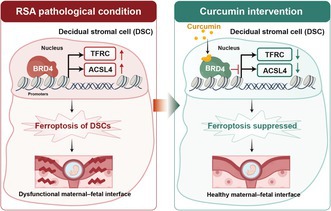

## 1 Introduction

Recurrent spontaneous abortion (RSA), defined as two or more consecutive pregnancy losses before 20 weeks of gestation, has emerged as a significant global reproductive health concern affecting approximately 1%–3% of reproductive‐age couples worldwide (Bender et al. 2023; Quenby et al. 2021). Despite advances in reproductive medicine, the underlying pathogenic mechanisms of RSA remain poorly defined in approximately 50% of cases, leaving many couples without targeted therapeutic options (Zafrilla et al. 2025). Current clinical approaches, primarily relying on progesterone supplementation, often show limited efficacy and inconsistent outcomes. This therapeutic bottleneck highlights an urgent need to shift toward safe, accessible, and non‐pharmacological strategies. Consequently, exploring interventions from the perspective of food science and nutrition has gained substantial traction, with increasing emphasis on utilizing dietary bioactive compounds to improve endometrial receptivity and pregnancy outcomes.

Among the various pathological factors contributing to RSA, impaired endometrial decidualization is recognized as a pivotal hallmark (Zhao et al. 2026; Guo et al. 2025). This intricate process, wherein endometrial stromal cells differentiate into specialized secretory cells, is indispensable for establishing maternal‐fetal immunotolerance (Li, Shi, et al. 2022; Xiong et al. 2024). Emerging evidence demonstrates that decidual dysfunction in RSA is critically driven by ferroptosis, an iron‐dependent form of regulated cell death involving lipid peroxidation (Dixon and Olzmann 2024; Sun et al. 2023). Pathological decidua from RSA patients exhibit a “metabolic storm” of local iron overload and polyunsaturated fatty acid enrichment, which overwhelms the endogenous antioxidant System Xc^−^/glutathione peroxidase 4 (GPX4) pathway (Wu et al. 2025) and subsequent structural collapse of DSCs (Su et al. 2025).

Crucially, cellular susceptibility to ferroptosis is tightly controlled by transcriptional regulation (Tang et al. 2021; Zhou et al. 2024). The bromodomain‐containing protein 4 (BRD4), a key epigenetic reader and transcriptional coactivator, has emerged as a critical regulator of ferroptosis‐related gene expression, including *TFRC* (iron transport) and *ACSL4* (lipid metabolism) (Zhu et al. 2024; Li, Yan, et al. 2024; Yang et al. 2022). Despite its established role in modulating ferroptosis in other contexts, whether BRD4 contributes to the pathological ferroptosis cascade in RSA‐associated decidual dysfunction remains completely unknown. This knowledge gap presents an opportunity to explore novel therapeutic strategies targeting epigenetic regulation in RSA.

Natural bioactive compounds derived from food sources have garnered increasing attention as potential therapeutic agents for reproductive disorders due to their favorable safety profiles and pleiotropic biological activities. Curcumin, a bioactive polyphenol derived from the rhizome of *Curcuma longa* , has been widely used as a dietary supplement due to its diverse pharmacological properties, including antioxidant, anti‐inflammatory, and epigenetic modulatory effects. In the context of reproductive health, curcumin has demonstrated protective effects against various disorders, including RSA (Saifi et al. 2022; Singh et al. 2022). However, while phenotypic studies suggest curcumin promotes decidualization and mitigates oxidative stress, its precise mechanism of action in RSA remains obscure (Xu et al. 2025). This ambiguity stems primarily from curcumin's pleiotropic nature, enabling interactions with multiple signaling pathways such as NF‐κB and Nrf2 (Bolger et al. 2022). While this multi‐target characteristic contributes to its broad health benefits, it also complicates the identification of specific molecular mechanisms underlying particular therapeutic effects, a challenge also encountered in mechanistic studies of complex bioactive herbal interventions (Song et al. 2023).

In this study, we integrated the classic CBA/J × DBA/2 mouse model of RSA with Limited Proteolysis‐Mass Spectrometry (LiP‐MS), a label‐free structural proteomics strategy, to map the direct interactome of Curcumin in human DSCs. This unbiased approach identified BRD4 as a primary physical target. We demonstrate that in RSA, pathological BRD4 occupancy at the promoters of *TFRC* and *ACSL4* drives a lethal ferroptotic cascade. Curcumin acts as a specific epigenetic modulator by binding to BRD4, thereby repressing pro‐ferroptotic drivers, restoring antioxidant defenses, and rescuing decidual competence.

Our work reveals that the BRD4‐regulated ferroptosis pathway represents a novel and druggable target for RSA and provides mechanistic evidence supporting the potential application of curcumin as a dietary intervention to improve pregnancy outcomes, providing scientific evidence for the potential application of curcumin as a dietary supplement in improving pregnancy outcomes.

## 2 Materials and Methods

### 2.1 Reagents and Antibodies

Curcumin (MedChemExpress, Cat. No.: HY‐N0005), Dydrogesterone (MedChemExpress, Cat. No.: HY‐B0257A), Erastin (MedChemExpress, Cat. No.: HY‐15763), JQ‐1 (MedChemExpress, Cat. No.: HY‐13030), 8‐bromoadenosine 3′, 5′‐cyclic monophosphate (8‐Br‐cAMP) (Sigma, Cat. No.: B5386), Medroxyprogesterone acetate (MPA) (APExBIO, Cat. No.: B1510), DMEM/F12 (Thermo Fisher, Cat. No.: 12634010), Fetal bovine serum (FBS) (Gibco, Cat. No.: 10099141); rLV‐CMV‐BRD4‐PGK‐Puro‐WPRE (BrainVTA Co. Ltd., Wuhan, China); CoraLite594‐Phalloidin (red) (Proteintech, Cat. No.: PF00003), FeRhoNox‐1 (Fe^2+^ indicator) (Maokangbio, Cat. No.: MX4558); mouse MDA ELISA kit (BYabscience, Cat. No.: BY‐WJZF0071), mouse SOD ELISA kit (BYabscience, Cat. No.: BY‐EM221637), mouse PRL ELISA kit (BYabscience, Cat. No.: BY‐EM220246), mouse IGFBP1 ELISA kit (BYabscience, Cat. No.: BY‐EM228061), human MDA ELISA kit (BYabscience, Cat. No.: BY‐WJZF0118), human SOD ELISA kit (BYabscience, Cat. No.: BY‐EH112140), human PRL ELISA kit (BYabscience, Cat. No.: BY‐EH110473), human IGFBP1 ELISA kit (BYabscience, Cat. No.: BY‐EH114674), GSH ELISA kit (BYabscience, Cat. No.: BY‐WJZF0366), GSSG ELISA kit (BYabscience, Cat. No.: BY‐WJZF1210). Antibodies: rabbit anti‐TFRC (Abcam, Cat. No.: ab214039), rabbit anti‐ACSL4 (Abcam, Cat. No.: ab155282), rabbit anti‐SLC7A11 (Abcam, Cat. No.: ab307601), mouse anti‐Vimentin (Abcam, Cat. No.: ab8978), rabbit anti‐BRD4 (Abcam, Cat. No.: ab243862), goat anti‐rabbit IgG H&L (Alexa Fluor 488) (Abcam, Cat. No.: ab150077), goat anti‐mouse IgG H&L (Alexa Fluor 647) (Abcam, Cat. No.: ab150115), goat anti‐mouse IgG H&L (Alexa Fluor 488) (Abcam, Cat. No.: ab150113).

### 2.2 Patients and Sample Collection

This study was conducted in compliance with the principles of the Declaration of Helsinki. Ethical approval was obtained from the Ethics Committee of Hangzhou Hospital of Traditional Chinese Medicine (Approval No. 2024KLL119). Written informed consent was acquired from all participants for the use of their clinical information. Decidua samples were collected from patients diagnosed with RSA according to established clinical criteria. Participants with known parental chromosomal abnormalities, uterine anomalies, endocrine disorders, antiphospholipid syndrome, autoimmune diseases, genital tract infections, or thrombophilia were excluded. Available clinical information was reviewed to ensure that the included samples met the predefined criteria for RSA‐related decidual tissue collection. These decidual tissues were used mainly for primary DSC isolation and mechanistic target‐discovery experiments.

### 2.3 Animals and Treatment

Eight‐ to ten‐week‐old female CBA/J mice (Huafukang Biotechnology) were mated with DBA/2 males (Huafukang Biotechnology) to establish the RSA model; age‐matched female CBA/J mice mated with BALB/c males (Huafukang Biotechnology) served as normal pregnancy controls. Mating was confirmed by the presence of a vaginal plug, designated as gestational day 1 (GD1). Pregnant mice were randomly divided into six groups: normal control (vehicle), RSA model, curcumin (20 mg/kg), curcumin (50 mg/kg), curcumin (100 mg/kg), and dydrogesterone (3.03 mg/kg, positive control). Curcumin was dissolved in 0.5% carboxymethylcellulose sodium. Daily oral gavage was initiated on GD1 and continued until sacrifice on GD8 (for decidual tissue analysis) or GD14 (for pregnancy outcome assessment). The animal experiments were approved by the IACUC at Zhejiang Chinese Medical University (No. IACUC‐202505‐13).

### 2.4 Cell Culture and Treatment

The telomerase‐immortalized human endometrial stromal cells (T‐hESCs) were used as a reproducible human endometrial stromal cell model suitable for decidualization induction. T‐hESCs were purchased from ATCC (Cat. No.: CRL‐4003). For in vitro decidualization experiments, T‐hESCs were seeded at a density of 2.5 × 10^4^ cells/cm^2^. Upon reaching approximately 70%–80% confluence, cells were treated with a decidualization cocktail containing 0.5 mM 8‐Br‐cAMP and 1 μM MPA for six days. The ferroptosis model was established by exposing decidualizing T‐hESCs to 10 μM Erastin for 24 h; for intervention experiments, cells received a treatment of curcumin (20, 50, 100 μM) or JQ1 (1 μM) during Erastin exposure.

### 2.5 Proteomic Analysis by Limited Proteolysis Mass Spectrometry (LiP‐MS)

Primary human DSCs were isolated from RSA endometrial tissues and cultured as described previously. Cells were lysed in PBS, and the lysates were aliquoted and treated with curcumin at 50 or 100 μM. Following incubation, limited proteolysis was carried out using protease K and trypsin. Peptide concentration was determined by OD_280_ measurement, and 2 μg of peptides from each sample were spiked with iRT standard peptides for normalization. Samples were analyzed by data‐independent acquisition (DIA) mass spectrometry. Peptides were separated on a UHPLC system and analyzed in positive ion mode on a high‐resolution mass spectrometer using DIA with 299 variable windows. DIA data were processed with Spectronaut, followed by protein clustering and domain analysis (Baker et al. 2024). Functional enrichment analyses (GO‐BP, KEGG) were performed using Metascape (https://metascape.org/) (Zhou et al. 2019).

### 2.6 An Integrative Bioinformatics Approach to Uncover BRD4 Downstream Targets in Ferroptosis

To identify ferroptosis‐related proteins, we retrieved relevant targets from the FerrDB database (http://www.zhounan.org/ferrdb/v3/pages/index.html) (Zhou et al. 2026). The overlapping proteins between the targets obtained from Lip‐MS and the ferroptosis‐related proteins were subjected to protein–protein interaction analysis (https://cn.string‐db.org/) (Szklarczyk et al. 2023).

To further explore the downstream targets of BRD4, we employed the hTFtarget database (https://guolab.wchscu.cn/hTFtarget/) to identify genes directly regulated by BRD4 (Zhang et al. 2020). These genes were then intersected with the ferroptosis‐related proteins. Finally, the resulting overlapping targets were analyzed using the MCODE plugin in Cytoscape to identify densely connected network modules.

### 2.7 Molecular Docking

The crystal structure of the BRD4 (PDB ID: 7REK) was retrieved from the Protein Data Bank (https://www.rcsb.org/) (Burley et al. 2025). The structure of curcumin was downloaded from the PubChem database (CID: 969516) (https://pubchem.ncbi.nlm.nih.gov/) (Kim et al. 2021). Molecular docking was performed using AutoDock Vina with default parameters. We utilized Discovery Studio Client for binding interaction analysis and PyMOL for the visualization of docking conformations.

### 2.8 Molecular Dynamics (MD) Simulations

The MD simulations were conducted with GROMACS 2022.3 (Lemkul 2024). Small molecule parameters, derived using the GAFF force field in AmberTools22, were integrated with RESP charges computed by Gaussian16W to construct the system topology. Following system construction and neutralization with NaCl, energy minimization was performed: first under the isothermal‐isovolumic (NVT) ensemble, then under the isothermal‐isobaric (NPT) ensemble, each comprising 100,000 steps (0.1 ps coupling time, 100 ps total). Finally, production MD runs were carried out under periodic boundary conditions for 100 ns at 26.85°C and 1.0 bar.

### 2.9 Immunofluorescence Staining

Decidual tissues were cut into 10 μm sections. T‐hESCs were seeded on glass coverslips and cultured until confluence. Tissue sections or cell slides were fixed with 4% paraformaldehyde for 15 min, permeabilized and blocked for 2 h. Samples were incubated with primary antibodies: Vimentin (1:200), TFRC (1:200), ACSL4 (1:200), and SLC7A11 (1:200). Then, samples were incubated with secondary antibodies (1:500) for 2 h. For Fe^2+^ detection, samples were incubated with FeRhoNox‐1 or Phalloidin for 30 min.

### 2.10 Transmission Electron Microscopy (TEM)

T‐hESCs were harvested and fixed with 2.5% glutaraldehyde for 2 h, then post‐fixed with 1% osmium tetroxide for 1 h. Cells were dehydrated through a graded acetone series (30%, 50%, 70%, 90%, 100%). The ultrastructure was examined using transmission electron microscopy (Hitachi, Tokyo, Japan).

### 2.11 Biochemical Assays

The concentrations of PRL, IGFBP1, Malondialdehyde (MDA), Superoxide dismutase (SOD), Glutathione (GSH), and oxidized glutathione (GSSG) in the decidual tissues or cells were quantified using ELISA kits. All assays were conducted following the manufacturer's instructions provided with the reagent kits. The optical density was measured at a wavelength of 450 nm using a microplate reader.

### 2.12 Chromatin Immunoprecipitation (ChIP)

Following fixation with formaldehyde (4°C, 12 min) and quenching with glycine (0.125 M), chromatin was isolated and fragmented via sonication. Immunoprecipitation was then conducted with an anti‐BRD4 antibody. The immunoprecipitated DNA was purified and eluted, followed by qPCR using SYBR Green Pro Taq HS Premix (AGBIO). Primer sequences for TFRC promoter: Forward: GGGATTACAAGTGTGGGCCA and reverse: AGTTCAAGATCAGCCTGGCC; ACSL4 promoter: Forward: TATCCTGGGGTGGAGTCCTG and reverse: TTCTGTCAGTCTCGCTGCTG.

### 2.13 Statistical Analysis

Data were analyzed with GraphPad Prism 9.0 and are presented as mean ± SEM. For comparisons among multiple groups, one‐way ANOVA, followed by Tukey's or Dunnett's test, was used as appropriate. Statistical significance was defined as *p* < 0.05. The number of biological replicates, animals, or independent experiments is indicated in the corresponding figure legends.

## 3 Results

### 3.1 Curcumin Ameliorates Decidualization Failure and Attenuates Embryo Resorption in RSA Mouse Models

**FIGURE 1:**
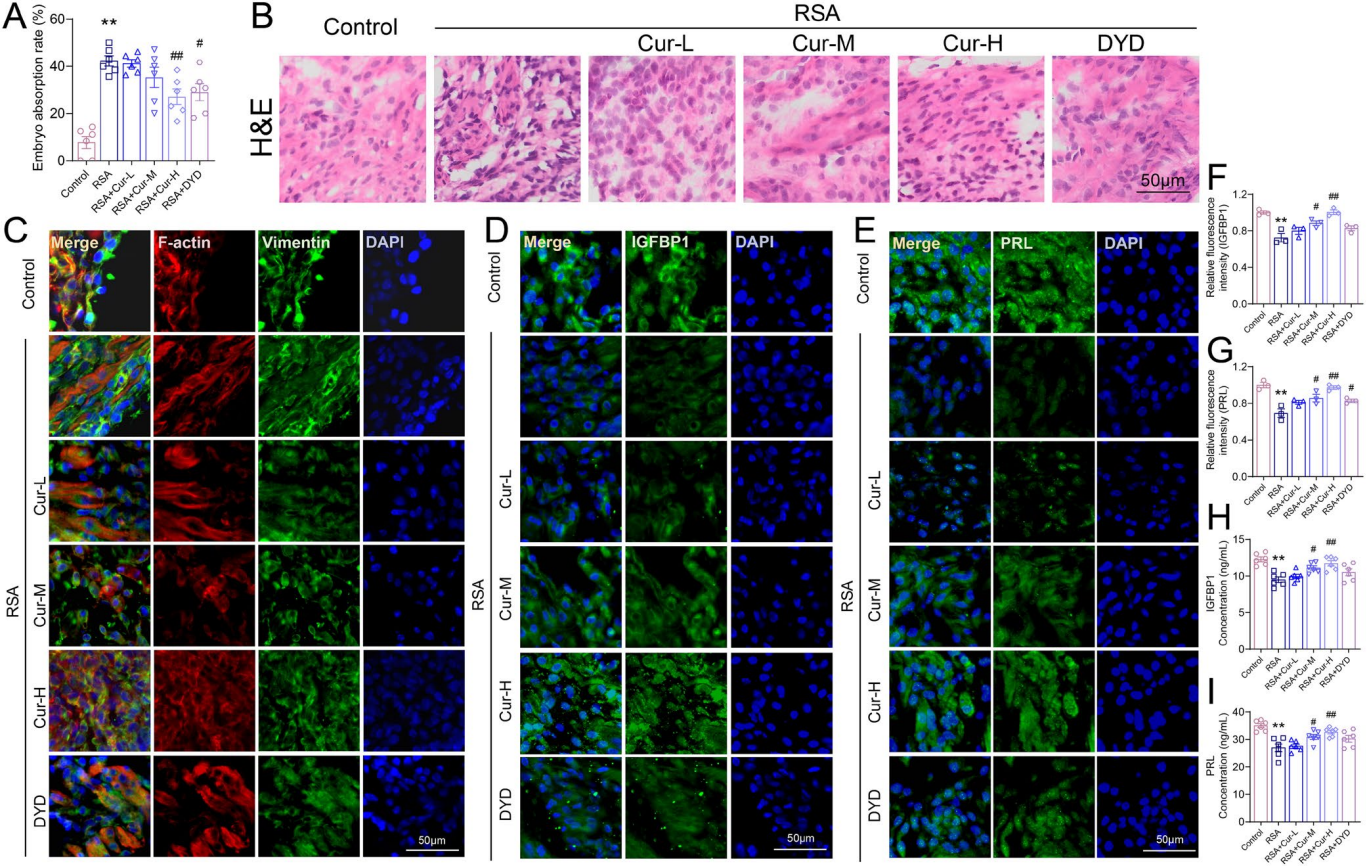
Curcumin ameliorates pregnancy outcomes and restores decidualization in the RSA mouse model. (A) Quantitative analysis of embryo resorption rates across different treatment groups (*n* = 6). (B) Representative hematoxylin and eosin (H&E) staining of decidual tissues (scale bar: 50 μm). (C) Immunofluorescence staining shows the organization of Factin (red) and vimentin (green) in decidual stromal cells; nuclei are counterstained with DAPI (blue) (scale bar: 50 μm). (D, E) Representative immunofluorescence staining of decidual markers IGFBP1 (D) and PRL (E) in decidual tissues (scale bar: 50 μm). (F, G) Quantification of IGFBP1 (F) and PRL (G) fluorescence intensity from images in D and E (*n* = 3). (H, I) ELISA was used to quantify the level of IGFBP1 (H) and PRL (I) in decidua tissues from each group (*n* = 6). Data are presented as means± SEM. Statistical significance is determined by one‐way ANOVA (Dunnett's post‐test). **p < 0.01 (normal control vs. RSA model); ^#^ *p* < 0.05, ^##^ *p* < 0.01 (treatment groups vs. RSA model group).

We established RSA model using CBA/J × DBA/2 mating combination. Curcumin was administered by oral gavage from gestational day 1 (GD1) through GD14. Pregnancy outcome assessment revealed a starkly elevated embryo resorption rate in the RSA model (*p* < 0.01). Curcumin intervention significantly attenuated fetal loss in a dose‐dependent manner. The high dose (100 mg/kg) reduced resorption to a level comparable to the clinical progestogen dydrogesterone, underscoring its potent efficacy (*p* < 0.05) (Figure 1A).

We next investigated whether this protective effect was mediated through improved endometrial remodeling. Histological analysis (H&E) of decidua on GD8 revealed severe structural disarray in RSA mice, featuring loose stromal compaction and inflammatory infiltration (Figure 1B). Immunofluorescence staining showed disorganized F‐actin stress fibers within Vimentin‐positive DSCs, indicative of cytoskeletal dysfunction. Curcumin treatment, particularly at the high dose, effectively restored both tissue architecture and cytoskeletal organization (Figure 1C). Consistent with these morphological improvements, immunofluorescence and ELISA results showed that the decidual markers (IGFBP1 and PRL), which were severely suppressed in the RSA group, were robustly rescued by curcumin (*p* < 0.05) (Figure 1D–I). Collectively, these data establish that curcumin acts directly on the endometrial stroma to restore the decidualization program, thereby preventing pregnancy failure.

### 3.2 Unbiased Chemoproteomics via LiP‐MS Identifies Ferroptosis as a Core Pathway Targeted by Curcumin

**FIGURE 2:**
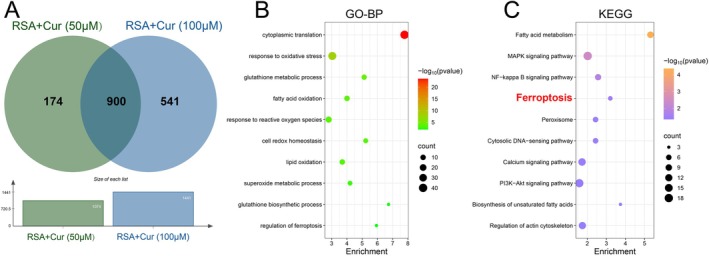
LiP‐MS identifies ferroptosis as a top pathway targeted by curcumin in human decidual stromal cells (DSCs). (A) Venn diagram shows the overlap of structurally altered proteins identified at the two concentrations. (B, C) Top Gene Ontology (GO) biological processes (B) and Kyoto Encyclopedia of Genes and Genomes (KEGG) pathways (C) enriched in the core curcumin‐interacting proteome (900 common proteins). Ferroptosis is the most significantly enriched KEGG pathway.

To map the direct molecular targets of curcumin within the decidual proteome, we employed LiP‐MS. This label‐free chemoproteomic strategy operates on the principle that small‐molecule binding alters a protein's susceptibility to proteolysis by inducing conformational changes or steric hindrance, thereby revealing ligand‐protein interactions with peptide‐level resolution. Lysates from primary human DSCs derived from RSA patients were incubated with curcumin (50 or 100 μM) or a vehicle control. LiP‐MS analysis identified a pronounced structural footprint, with 1074 and 1441 structurally altered proteins in the medium‐ and high‐dose groups, respectively. A core high‐confidence interactome of 900 proteins was common to both treatment concentrations (Figure 2A).

GO enrichment analysis of this core interactome highlighted processes integral to oxidative stress response and lipid metabolism, including “cell redox homeostasis” and “fatty acid oxidation” (Figure 2B). Strikingly, KEGG pathway analysis revealed enrichment of pathways including “Ferroptosis” and “NF‐kappa B signaling pathway” (Figure 2C). This unbiased proteomic profiling provides direct, systematic evidence that curcumin's pharmacological activity in the decidua converges on the regulatory machinery of ferroptosis.

### 3.3 Curcumin Suppresses Ferroptosis and Restores Decidual Function In Vivo and In Vitro

**FIGURE 3:**
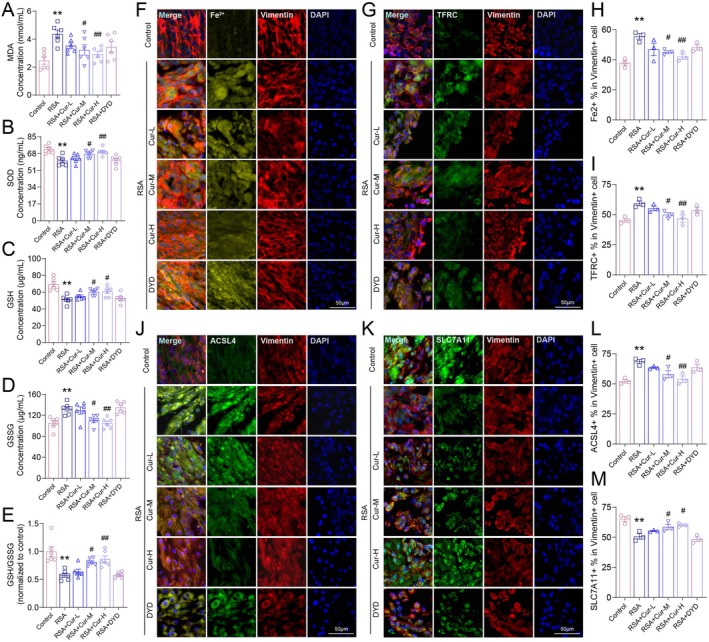
Curcumin suppresses ferroptosis in the decidua of RSA mice. (A–E). Biochemical assessment of oxidative stress markers in decidual tissues on GD8: Malondialdehyde (MDA) content (A), superoxide dismutase (SOD) activity (B), glutathione (reduced form) level (C), glutathione disulfide level (D), and the reduced/oxidized glutathione (GSH/GSSG) ratio (E) (*n* = 6). (F–I) IF co‐staining was used to detect Fe^2+^ and TFRC in vimentin‐positive cells from different group mice (*n* = 3). Scale bar: 50 μm. (J–M) IF co‐staining was used to detect ACSL4 and SLC7A11 in vimentin‐positive cells from different group mice (*n* = 3). Scale bar: 50 μm. Data are presented as means ± SEM. Statistical significance is determined by one‐way ANOVA (Dunnett's post‐test). **p < 0.01 (normal control vs. RSA model); ^#^ *p* < 0.05, ^##^ *p* < 0.01 (treatment groups vs. RSA model group).

Guided by the proteomic prediction, we validated the inhibitory effect of curcumin on ferroptosis using complementary in vivo and in vitro models. In decidual tissues from RSA mice, biochemical analysis revealed hallmark features of ferroptosis. Compared to controls, the RSA model group exhibited a significant increase in MDA levels (*p* < 0.01) (Figure 3A), accompanied by decreased SOD activity (*p* < 0.01) (Figure 3B) and a disrupted glutathione redox balance (GSH/GSSG ratio) (*p* < 0.01) (Figure 3C–E). Curcumin treatment dose‐dependently reversed these alterations, with medium and high doses significantly reducing MDA levels, restoring SOD activity and the GSH/GSSG ratio (vs. model group, *p* < 0.05) (Figure 3A–E). Immunofluorescence staining provided spatial confirmation of ferroptotic stress within the decidua. Vimentin‐positive stromal cells in the RSA group showed pronounced intracellular ferrous iron (Fe^2+^) accumulation (*p* < 0.01) (Figure 3F,H), elevated expression of the ferroptosis drivers transferrin receptor (TFRC) (*p* < 0.01) (Figure 3G,I) and acyl‐CoA synthetase long‐chain family member 4 (ACSL4) (*p* < 0.01) (Figure 3J,L), and concurrent downregulation of the suppressor SLC7A11 (*p* < 0.01) (Figure 3K,M). Curcumin intervention ameliorated these molecular markers (*p* < 0.05).

**FIGURE 4:**
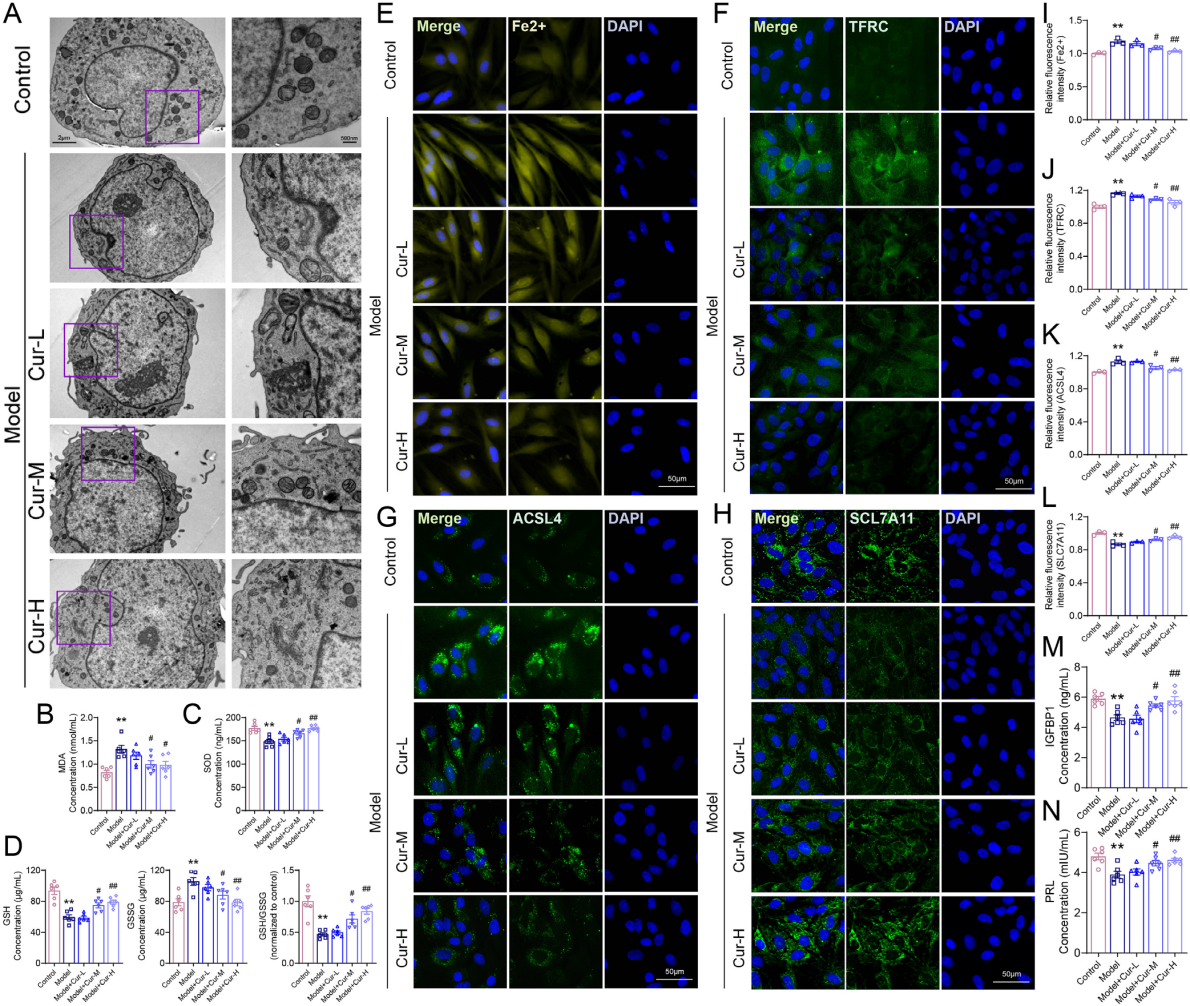
Curcumin rescues Erastin induced ferroptosis and restores decidualization in telomerase‐immortalized human endometrial stromal cells (T hESCs). (A) Representative transmission electron microscopy (TEM) images of mitochondrial ultrastructure in T hESCs under indicated treatments indicated shrunken mitochondria with condensed membranes, characteristic of ferroptosis. (B–D) Biochemical assessment of ferroptosis markers: Malondialdehyde (MDA) content (B), superoxide dismutase (SOD) activity (C), and the reduced/oxidized glutathione (GSH/GSSG) ratio (D) (*n* = 6). (E–H) Representative immunofluorescence images showing intracellular ferrous iron (Fe^2+^) (E), and expression of TFRC (F), ACSL4 (G), and SLC7A11 (H) (scale bar: 50 μm). (I–L) Quantification of fluorescence intensity for Fe^2+^ (I), TFRC (J), ACSL4 (K), and SLC7A11 (L) (*n* = 3). (M, N) ELISA was used to quantify the level of IGFBP1 (M) and PRL (N) from each group (*n* = 6). Data are presented as means ± SEM. Statistical significance is determined by one‐way ANOVA (Dunnett's post‐test). **p < 0.01 (normal control vs. model group); ^#^ *p* < 0.05, ^##^ *p* < 0.01 (treatment groups vs. model group).

In vitro, we challenged decidualizing T‐hESCs with the ferroptosis inducer Erastin (10 μM). TEM revealed the classical ultrastructural signature of ferroptosis in Erastin‐treated cells, including mitochondrial shrinkage, increased membrane density (Figure 4A). Co‐treatment with curcumin (100 μM) substantially preserved mitochondrial integrity. Furthermore, curcumin significantly attenuated Erastin‐induced accumulation of MDA, restored SOD activity, and corrected the impaired GSH/GSSG ratio (all *p* < 0.01 vs. Erastin group) (Figure 4B–D). Biochemically, curcumin normalized the Erastin‐induced dysregulation of intracellular Fe^2+^ (Figure 4E,I), TFRC (Figure 4F,J), ACSL4 (Figure 4G,K), and SLC7A11 (all *p* < 0.01 vs. Model group) (Figure 4H,L). Critically, this pharmacological suppression of ferroptosis was functionally consequential. Curcumin co‐treatment rescued IGFBP1 and PRL, which were severely suppressed by Erastin (*p* < 0.05) (Figure 4M,N). These data demonstrate that inhibition of the ferroptotic pathway is sufficient to restore the decidualization capacity of stromal cells, establishing it as a critical mechanism through which curcumin exerts its therapeutic effect.

### 3.4 Molecular Docking and Molecular Dynamics Simulations Reveal That Curcumin Binds to BRD4

**FIGURE 5:**
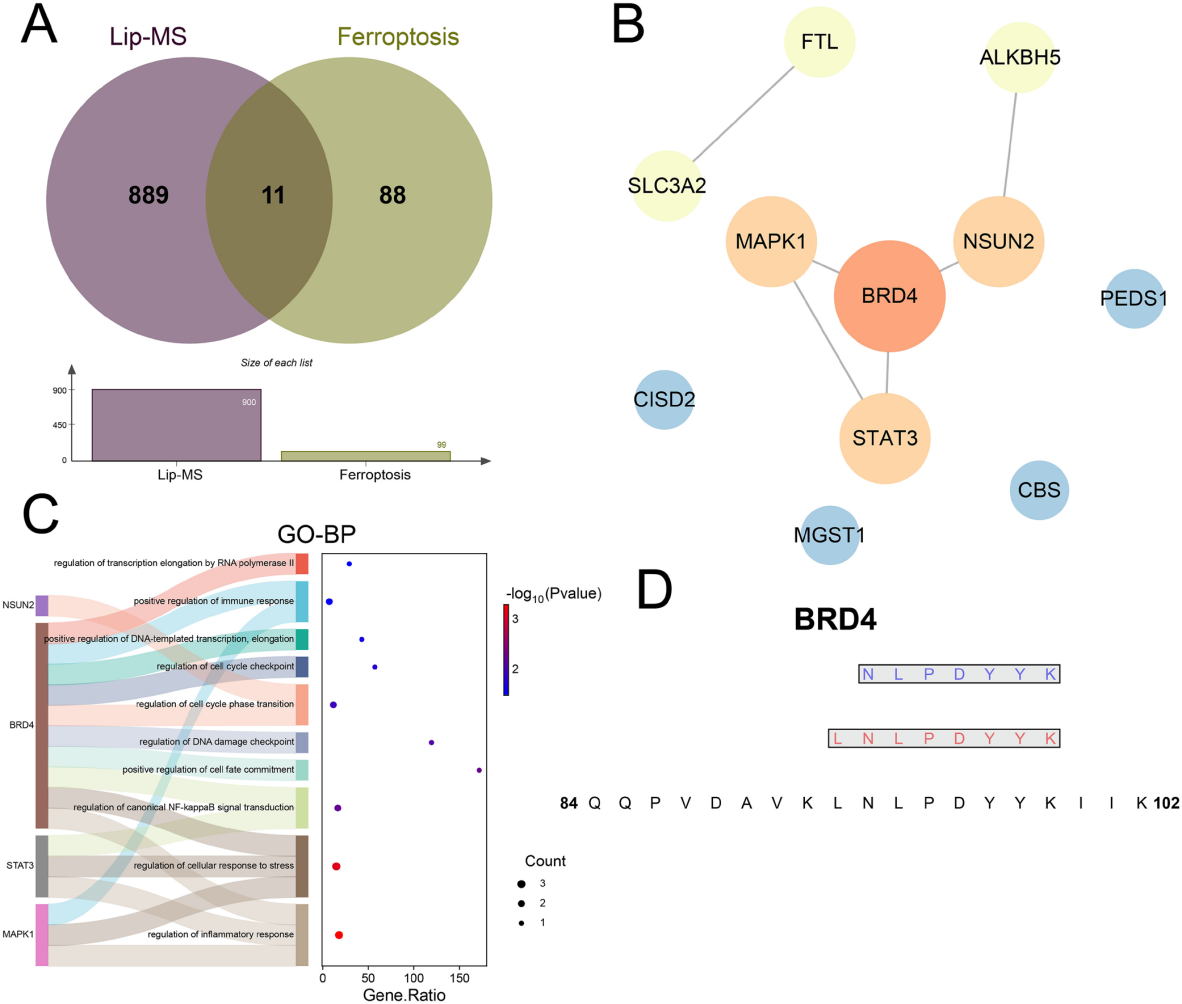
Bioinformatic screening identifies BRD4 as a central node linking curcumin binding to ferroptosis regulation. (A) Venn diagram showing the intersection between the core curcumin interacting proteome (900 proteins) and the ferroptosis‐related protein database (FerrDB), yielding 11 candidate targets. (B) Protein–protein interaction (PPI) network of the 11 overlapping proteins, with BRD4 highlighted as a central hub. (C) GO‐BP enrichment analysis of the 11 candidate proteins, highlighting terms related to transcriptional regulation. (D) Assessment of BRD4 druggability based on characteristic peptide segments.

To identify the upstream target mediating curcumin's inhibition of ferroptosis, we intersected its 900 core binding proteins with the ferroptosis database FerrDB, yielding 11 candidates (Figure 5A). Protein–protein interaction network analysis pinpointed Bromodomain‐containing protein 4 (BRD4) as a central hub (Figure 5B). Gene Ontology analysis of these candidates highlighted enrichment in “transcriptional elongation” and “cell fate commitment”, processes coherent with BRD4's canonical role as an epigenetic reader (Figure 5C). Based on the results from LiP‐MS, key peptide segments within the BRD4 protein structure were identified. LiP‐MS identified key peptide segments in BRD4 as structured, accessible, and functional regions, making them prime pharmacological targets (Figure 5D).

**FIGURE 6:**
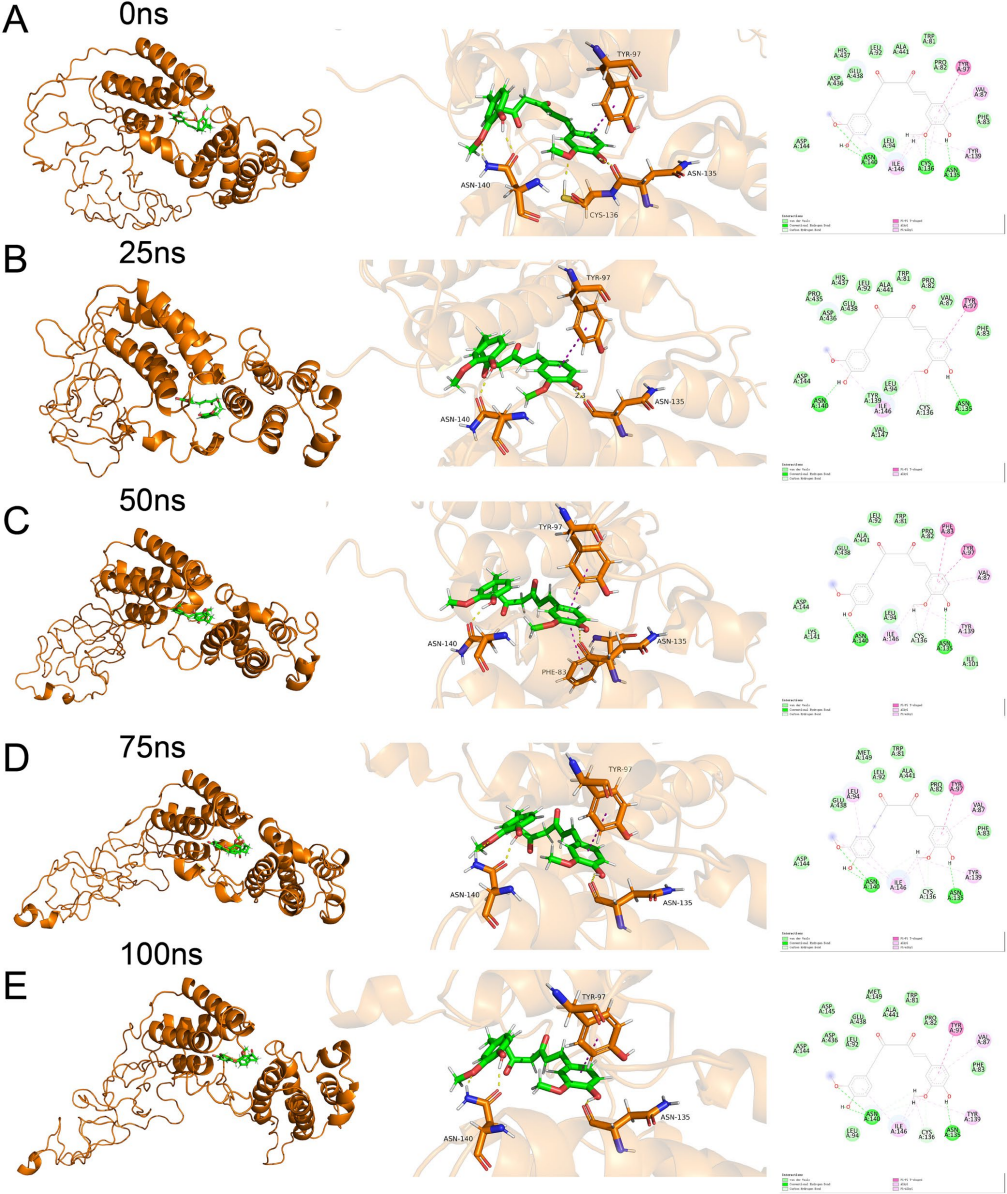
Molecular docking identifies the binding pose of curcumin within the BRD4. The molecular docking results of curcumin with BRD4 were validated. The figure depicts representative binding poses extracted from the molecular dynamics simulation at key time points: 0 ns (A), 25 ns (B), 50 ns (C), 75 ns (D), and 100 ns (E).

**FIGURE 7:**
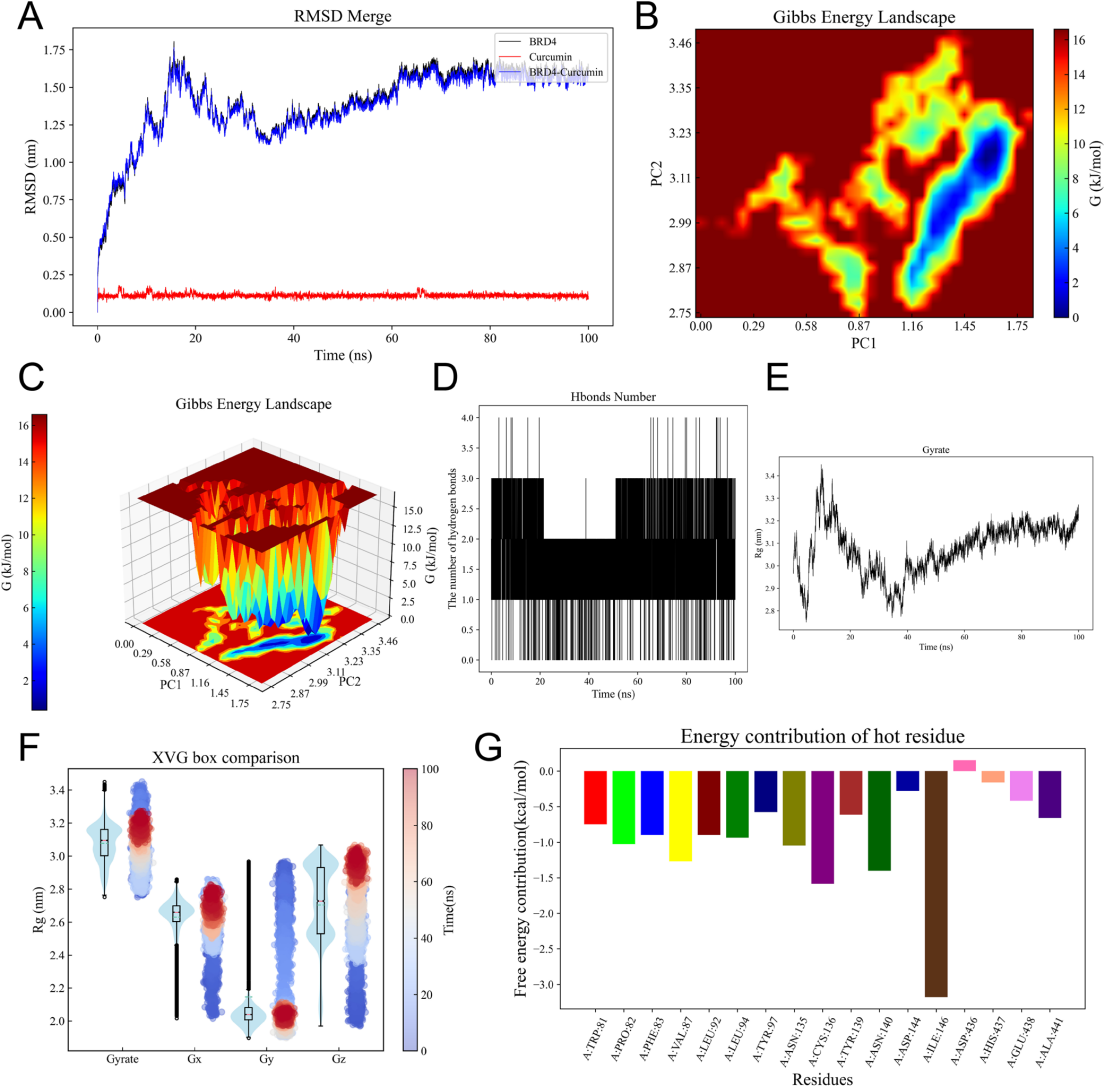
Molecular dynamics simulations confirm the stability of the curcumin BRD4 complex. (A) Root mean square deviation (RMSD) of the BRD4 backbone during the 100 ns simulation. (B, C) Two‐dimensional (B) and three‐dimensional (C) Gibbs free energy landscapes for the complex between curcumin and BRD4. (D) The number of hydrogen bonds between curcumin and BRD4 over the simulation time. (E) Radius of gyration (Rg) profile of the BRD4‐curcumin complex throughout the 100‐ns simulation. (F) Rg boxplot of the curcumin‐BRD4 complex. (G) Per‐residue energy decomposition identifying ILE146 as the major stabilizing residue.

Molecular docking simulations were performed to identify the binding site of curcumin in BRD4. Analysis of snapshots taken at 0, 25, 50, 75, and 100 ns showed that ILE146 consistently formed hydrogen bonds with curcumin across all time points (Figure 6). Subsequent 100‐ns molecular dynamics simulations demonstrated that the curcumin‐BRD4 complex achieved a stable conformation, with the protein backbone root‐mean‐square deviation (RMSD) plateauing around 70 ns after the initial equilibration phase (Figure 7A). In addition, the Gibbs free energy was precisely computed on the basis of the RMSD and Rg values of the complex. As illustrated in Figure 7B,C, these results verify that the complex maintains a stable thermodynamic state. The number of hydrogen bonds in the curcumin‐BRD4 complex ranged from 2 to 4 (Figure 7D), and such a high hydrogen bond density indicates a strong interaction between BRD4 and curcumin. The free energy landscape (FEL), constructed from RMSD and Rg data, demonstrates that BRD4 adopts a stable conformation at the energy minimum cluster (Figure 7E,F).

Furthermore, to quantify the binding stability between curcumin and the BRD4 protein receptor, the binding free energy and residue‐specific energy contributions were calculated via the molecular mechanics‐generalized born surface area (MM‐GBSA) method. The outcomes of per‐residue energy decomposition are displayed in Figure 7G. As presented in Table 1, the computed binding free energy of the curcumin‐BRD4 complex was −31.63 ± 1.82 kcal/mol, which reflects a high binding affinity. Taken together, these findings suggest that ILE146 probably exerts considerable binding affinity for curcumin.

**TABLE 1:** MM‐GBSA calculation results of curcumin‐BRD4 complex (mean ± SEM).

Contribution components	BRD4‐Curcumin (kcal/mol)
Δ_VDWAALS_	−40.19 ± 0.46
Δ*E*_elec_	−22.73 ± 1.71
Δ*E*_GB_	37.34 ± 0.42
Δ*E*_surf_	−6.04 ± 0.11
Δ*G*_gas_	−62.92 ± 1.77
Δ*G*_solvation_	31.29 ± 0.43
ΔTotal	−31.63 ± 1.82

### 3.5 BRD4 Is Required for Curcumin‐Mediated Suppression of Ferroptosis and Restoration of Decidualization

**FIGURE 8:**
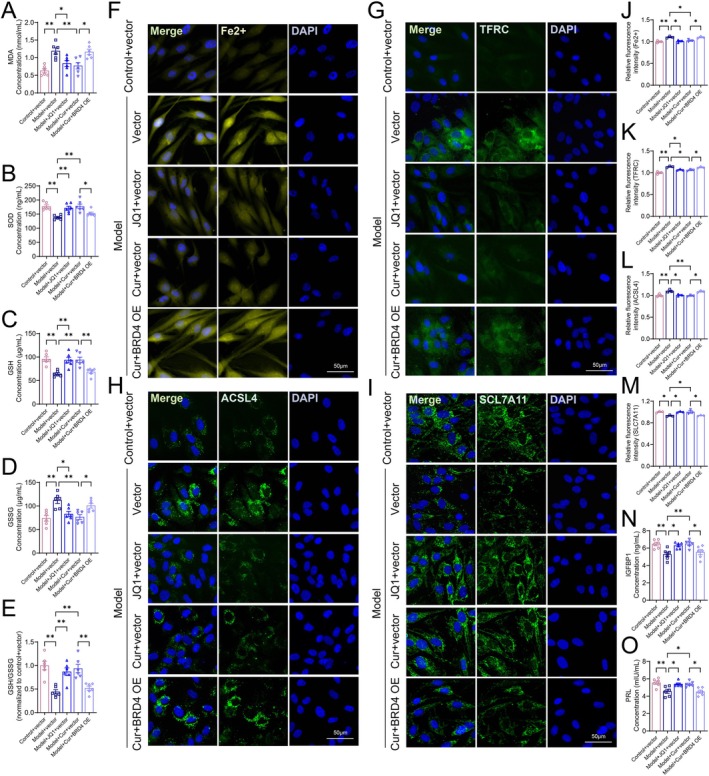
BRD4 is a necessary effector for curcumin's suppression of ferroptosis and rescue of decidualization. (A–E) Biochemical analysis in T hESCs showing the effects of BRD4 inhibition (JQ1) and overexpression on ferroptosis markers: MDA content (A), SOD activity (B), GSH level (C), GSSG level (D), and GSH/GSSG ratio (E) (*n* = 6). (F–M) Representative immunofluorescence images (F–I) and quantification (J–M) of intracellular Fe^2+^ (F, J), TFRC (G, K), ACSL4 (H, L), and SLC7A11 (I, M) expression (scale bar: 50 μm) (*n* = 3). (N, O) ELISA was used to quantify the level of IGFBP1 (N) and PRL (O) from each group (*n* = 6). Data are presented as means ± SEM. Statistical significance is determined by one‐way ANOVA (Tukey post‐test). *p < 0.05, **p < 0.01.

We next assessed the functional hierarchy of BRD4 within the ferroptosis pathway. In T‐hESCs subjected to Erastin‐induced ferroptotic stress, chemical inhibition of BRD4 with JQ1 phenocopied the effects of curcumin (100 μM), which was manifested by reduced MDA levels, increased SOD activity, elevated GSH/GSSG ratio (*p* < 0.05) (Figure 8A–E), decreased Fe^2+^ levels, downregulated TFRC expression, reduced ACSL4 expression, and upregulated SLC7A11 expression (*p* < 0.05) (Figure 8F–M). Meanwhile, IGFBP1 and PRL levels were increased (*p* < 0.05) (Figure 8N,O). Conversely, overexpression of BRD4 attenuated curcumin's capacity to inhibit ferroptosis and rescue impaired decidualization, thereby establishing BRD4 as a necessary downstream effector (Figure 8).

**FIGURE 9:**
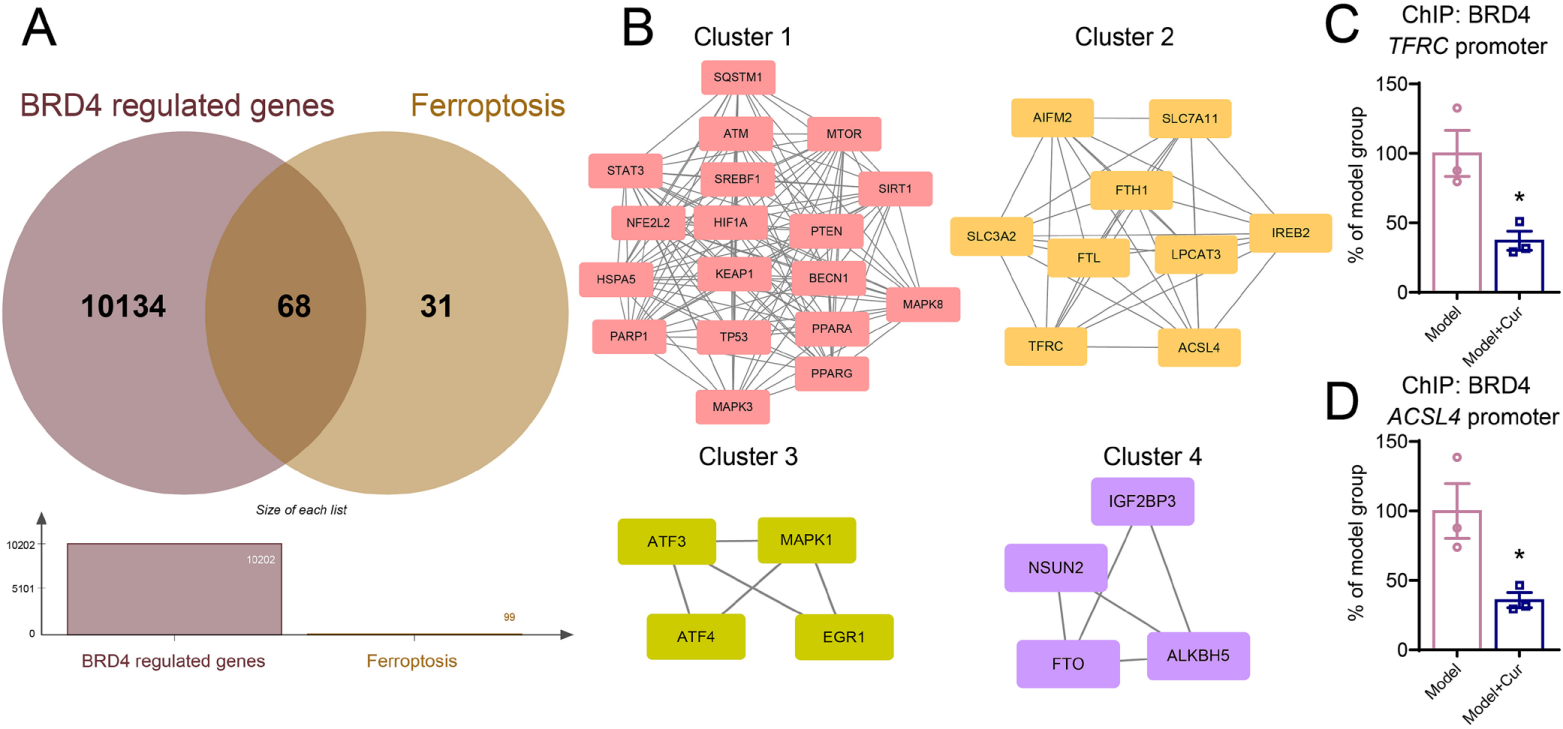
BRD4 transcriptionally regulates pro ferroptotic genes TFRC and ACSL4, which is antagonized by curcumin. (A) Venn diagram showing the overlap between BRD4 potential target genes (from hTFtarget database) and known ferroptosis‐related genes, identifying 68 common targets. (B) MCODE analysis of the 68 overlapping genes, revealing four functional clusters. Cluster 2 is most significantly associated with ferroptosis regulation and contains TFRC and ACSL4. (C, D) Chromatin immunoprecipitation quantitative PCR (ChIP qPCR) analysis showing BRD4 occupancy at the promoter regions of TFRC (C) and ACSL4 (D) in T hESCs. Curcumin treatment significantly reduces BRD4 binding (*n* = 3). Data are presented as means ± SEM. Statistical significance is determined by two‐tailed Student's *t*‐test. *p < 0.05 versus model group.

To delineate the downstream transcriptional targets of BRD4 in decidual ferroptosis, we first performed a systematic bioinformatic screening. Using the hTFtarget database, we identified 10,202 genes potentially directly regulated by BRD4. Intersection of this list with 99 known ferroptosis‐related proteins yielded 68 overlapping targets (Figure 9A). Functional clustering of these 68 genes by MCODE analysis revealed four distinct modules, among which Cluster 2 was most significantly associated with ferroptosis regulation (Figure 9B). This analysis highlighted TFRC and ACSL4 as core components of the BRD4‐driven pro‐ferroptotic network.

We therefore hypothesized that curcumin exerts its effect by antagonizing BRD4's transcriptional activity at specific chromatin loci. Chromatin immunoprecipitation‐quantitative PCR (ChIP‐qPCR) confirmed significant enrichment of BRD4 at the promoter regions of TFRC and ACSL4. Critically, curcumin treatment substantially reduced BRD4 occupancy at these promoters (*p* < 0.05), which correlated with suppressed transcription and protein expression of both genes (Figure 9C,D). These data establish that curcumin functions as a BRD4 antagonist to repress a pro‐ferroptotic transcriptional program and thereby restore cell viability and function.

## 4 Discussion

As dietary polyphenols gain traction as non‐pharmacological interventions for reproductive health, understanding their precise mechanisms of action is paramount. Although curcumin has long been recognized for its potential to improve pregnancy outcomes, the lack of a defined molecular target in the context of RSA has significantly hindered its clinical translation (Ahamed et al. 2022; Liu, Zhou, et al. 2023). This study delineates an integrated mechanism through which curcumin counteracts recurrent spontaneous abortion. We first demonstrate its ability to restore decidualization and pregnancy outcomes in RSA mice. Employing an unbiased chemoproteomic strategy, we then identified ferroptosis as the central pathological pathway targeted by curcumin in human decidual cells, which was functionally validated across models. Subsequent intersectional analysis converged on the epigenetic reader BRD4 as the critical link between curcumin binding and ferroptosis regulation. We establish that curcumin represses this pathological transcriptional program by directly binding to BRD4 and displacing it from chromatin. Mechanistically, this pathological transcriptional program is driven by BRD4 occupancy at the promoters of the pro‐ferroptotic genes *TFRC* and *ACSL4*, which leads to decidual dysfunction. Collectively, these findings reposition curcumin from a pleiotropic natural product into a defined epigenetic modulator that antagonizes BRD4 to suppress ferroptosis, unveiling a novel therapeutic axis for RSA.

The protective effects of dietary bioactive compounds against reproductive disorders have garnered increasing attention in the field of food science and nutrition. Natural polyphenols, including curcumin, resveratrol, and quercetin, have demonstrated beneficial effects on female reproductive health through their antioxidant and anti‐inflammatory properties (Chang et al. 2025; Li, Li, et al. 2024). During pregnancy, oxidative stress at the maternal‐fetal interface contributes to various complications, including preeclampsia, intrauterine growth restriction, and spontaneous abortion (Dos et al. 2022). Curcumin, as a pleiotropic natural polyphenol, has been reported to interact with multiple molecular targets in various biological systems (Li, Zhu, et al. 2022). However, its precise mechanism of action in recurrent spontaneous abortion remains poorly defined, and most previous studies have attributed its protective effects to generalized antioxidant or anti‐inflammatory activities. This lack of context‐specific mechanistic clarity has significantly hindered its clinical translation as a targeted dietary intervention for reproductive health. Our study addresses this critical gap by identifying BRD4 as a direct physical target of curcumin in human decidual stromal cells. Using LiP‐MS chemoproteomics, we identified BRD4 as a primary physical target within the native decidual proteome. Functional assays confirmed that BRD4 inhibition recapitulated all core effects of curcumin and restoration of decidual marker expression. Conversely, BRD4 overexpression substantially attenuated these protective effects. These findings establish BRD4 inhibition as a central mechanism underlying curcumin's protective effects in RSA. This identification of a context‐specific epigenetic target not only removes a critical barrier to the clinical translation of curcumin, but also sets the stage for investigating the cell‐type‐specific mechanisms through which it exerts its protective effects.

The context specificity of this curcumin‐BRD4 interaction unveils a previously unrecognized vulnerability in the decidua of RSA. Previous studies have reported that curcumin activates the Nrf2/HO‐1 pathway to counteract oxidative stress and ferroptosis (Ashrafizadeh et al. 2020; Zhu et al. 2025). Our work extends these findings by identifying BRD4 as a direct physical target of curcumin, which transcriptionally suppresses pro‐ferroptotic genes *TFRC* and *ACSL4*. The Nrf2 pathway primarily enhances antioxidant capacity (e.g., SLC7A11/GPX4), whereas BRD4 inhibition directly curtails iron uptake and lipid peroxidation machinery. Thus, curcumin likely acts through convergent, synergistic pathways—a direct epigenetic modulation of BRD4 and an indirect activation of Nrf2—to restore decidual redox homeostasis. Interestingly, BRD4's role in ferroptosis is not universal but dictated by cellular lineage. In certain cancers, BRD4 inhibition can induce ferroptosis (Zhao et al. 2025; Liu, Chen, et al. 2023; Dong et al. 2026), indicating an anti‐ferroptotic function. Conversely, in other cellular contexts, BRD4 promotes ferroptosis (Yang et al. 2022). In line with this, our data reveal a pro‐ferroptotic, pathology‐driving role for BRD4 in decidual cells, where it transcriptionally amplifies a deleterious ferroptotic program. Curcumin acts as a natural epigenetic modulator that directly targets BRD4 to normalize the pathological transcriptomic profile in dysfunctional decidua. This explains why curcumin treatment precisely “tunes down” the overexpression of *TFRC* and *ACSL4* without global shutdown of essential housekeeping genes, offering a potential therapeutic window superior to broader epigenetic inhibitors.

Beyond its direct effects on decidual cell survival and function, BRD4‐mediated ferroptosis also has profound implications for the immune microenvironment at the maternal‐fetal interface. Although impaired immune tolerance at the maternal‐fetal interface is widely recognized as the core pathological basis of the CBA/J × DBA/2 mouse model of recurrent spontaneous abortion (RSA), the role of decidual stromal cells (DSCs) has long been confined to providing structural support for embryo implantation, while their critical function in actively shaping the maternal‐fetal immune microenvironment remains underappreciated. Recent studies have fundamentally revised this traditional view and established DSCs as active architects and central regulators of the maternal‐fetal immune microenvironment. Single‐cell spatial transcriptomic analysis in early pregnant mouse decidua has revealed that DSCs participate in constructing the immune microenvironment by forming functional decidual hubs. Among these, dysfunction of a subset of immune‐featured DSCs (iDSCs) can directly lead to pregnancy failure (Yang et al. 2023). Clinical evidence further confirms that distinct dysfunctional DSC subsets exist in the decidua of RSA patients. Such aberrant DSCs disrupt decidual immune homeostasis and interfere with the differentiation of regulatory T cells, ultimately breaking maternal‐fetal immune tolerance and driving adverse pregnancy outcomes (Qin et al. 2024). Collectively, these studies support a central causal relationship in which DSC dysfunction drives immune dysregulation in RSA.

Findings from the present study provide important insights into the upstream triggers underlying this causal relationship. We observed extensive ferroptosis in DSCs in the RSA mouse model, resulting in severe disruption of decidual tissue structure. Based on the above evidence, we propose that ferroptosis may directly reduce the number of immunomodulatory DSC subsets and exacerbate immune microenvironment imbalance. By inhibiting BRD4‐mediated ferroptosis, curcumin not only rescues DSC survival and decidualization but also preserves the integrity of DSCs as immune regulatory hubs, indirectly restoring immune homeostasis at the maternal‐fetal interface. These findings deepen the mechanistic understanding of RSA pathogenesis and provide a theoretical basis for developing therapeutic strategies that jointly target ferroptosis and immune regulation.

Beyond the specific context of RSA, our findings align with the broader paradigm that stromal cells within local microenvironments play pivotal roles in maintaining tissue homeostasis and dictating disease outcomes. For instance, mesenchymal stromal cells can profoundly remodel the immune microenvironment (Zhang et al. 2024), and stromal cell‐derived secretory proteins have been shown to critically mediate cellular interactions and disease progression in cancer (Cao et al. 2026). Analogous to these pathological contexts, our study demonstrates that maintaining endometrial stromal cell integrity by blocking the pro‐ferroptotic BRD4 axis is indispensable for overcoming decidual dysfunction. This cross‐disease parallel suggests that targeting stromal cell vulnerabilities may represent a broadly applicable therapeutic strategy.

These findings provide preliminary but meaningful insights into the translational potential of curcumin as a dietary intervention for reproductive health. By elucidating the molecular mechanism underlying curcumin's protective effects against RSA, our work establishes a tentative mechanistic basis to support its potential clinical application. Accumulating clinical data suggest a generally favorable safety and tolerability profile of curcumin supplementation (Filardi et al. 2020), which further supports its tentative use during the peri‐conception period. Beyond intervention efficacy, the BRD4‐ferroptosis axis characterized here may also act as a candidate signature to guide patient stratification. RSA patients with elevated decidual expression of this axis are likely to respond better to curcumin or subsequent BRD4‐targeted treatments. In addition, our results indicate that moderate modulation of aberrant transcriptional programs, instead of full pathway suppression, helps restore endometrial homeostasis. This observation offers a practical reference for developing mild nutritional strategies to manage RSA.

Several limitations should be acknowledged. First, although our data support a BRD4‐centered mechanism, the upstream signals responsible for BRD4 activation in RSA remain unclear. Future studies should investigate whether hormonal imbalances, oxidative stress, or other maternal factors drive BRD4 dysregulation. Second, although the CBA/J × DBA/2 model is widely used for studying RSA‐related pregnancy failure, no animal model can fully recapitulate the heterogeneity of human RSA. Further studies in humanized models or clinical samples are warranted. Third, the curcumin bioavailability remains a recognized challenge including sustained‐release or advanced delivery systems to improve tissue exposure (Ji et al. 2023), and further define the optimal dosing, timing, and safety of curcumin supplementation during the peri‐conception period before clinical translation.

In conclusion, we have delineated a coherent and druggable pathway wherein curcumin exerts its therapeutic effect against RSA by directly antagonizing BRD4 to suppress a deleterious ferroptotic program in decidual stromal cells. This work not only clarifies the long‐obscured mechanism of a promising natural compound but also illuminates BRD4‐mediated transcriptional control of ferroptosis as a fundamental regulatory node in endometrial receptivity. These findings provide scientific evidence supporting the potential application of curcumin as a dietary supplement for improving pregnancy outcomes, providing a theoretical basis for nutritional intervention in RSA.

## Author Contributions

**Yiming Ma:** conceptualization, methodology, validation, visualization, writing – original draft, writing – review and editing. **Weiping Chen:** conceptualization, writing – original draft, validation, methodology. **Tian Xie:** conceptualization, investigation, validation, formal analysis, software. **Yueling Wu:** conceptualization, methodology, validation, software. **Yangyang Xing:** conceptualization, methodology, software, visualization. **Xiaoxuan Zhao:** conceptualization, investigation, funding acquisition, writing – original draft, writing – review and editing, supervision. **Jialin He:** conceptualization, investigation, supervision, resources, project administration, writing – review and editing.

## Funding

This research was funded by the Research Project of Zhejiang Chinese Medical University, China (Grant 2026JKZKTS60), Zhejiang Traditional Medicine and Technology Program, China (Grant 2025ZR173), Medical Scientific Research Foundation of Zhejiang Province, China (Grant 2025KY158) to X.Z. Hangzhou Medical Key Discipline (Peak Discipline) (Grant 2025HZGF11) Zhejiang Province “Small and Strong” Clinical Innovation Team (Ovarian Insufficiency Infertility “Small and Strong” Clinical Innovation Team) (Grant CXTD202501052). Zhejiang Clinical Medical Research Center for Gynecological Diseases (Grant 2022E5002). National Traditional Chinese Medicine Advantage Specialty: Traditional Chinese Medicine Gynecology. Zhejiang Province Traditional Chinese Medicine Advantage Specialty Alliance (Gynecology) (Grant 2025HZLM02). Hangzhou Traditional Chinese Medicine Gynecology Specialty Alliance (Grant 2025HZZKLM01). Research Project of Zhejiang Chinese Medical University, China (Grant 2026JKZDZC09).

## Ethics Statement

This study was conducted in compliance with the principles of the Declaration of Helsinki. Ethical approval was obtained from the Ethics Committee of Hangzhou Hospital of Traditional Chinese Medicine (Approval No. 2024KLL119). Written informed consent was acquired from all participants for the use of their clinical information. The animal experiments were approved by the IACUC at Zhejiang Chinese Medical University (No. IACUC‐202505‐13).

## Conflicts of Interest

The authors declare no conflicts of interest.

## Data Availability

The raw sequence data reported in this paper have been deposited in the Genome Sequence Archive in National Genomics Data Center, China National Center for Bioinformation/Beijing Institute of Genomics, Chinese Academy of Sciences (OMIX018785) that are accessible at https://ngdc.cncb.ac.cn/.
